# Fattening Pigs with Tannin-Rich Source (*Ceratonia siliqua* L.) and High Doses of Vitamin E: Effects on Growth Performance, Economics, Digestibility, Physiology, and Behaviour

**DOI:** 10.3390/ani14131855

**Published:** 2024-06-22

**Authors:** Diego Nicolas Bottegal, María Ángeles Latorre, Sandra Lobón, Marçal Verdú, Javier Álvarez-Rodríguez

**Affiliations:** 1 Departament de Ciència Animal, Universitat de Lleida, Av. Alcalde Rovira Roure 191, 25198 Lleida, Spain; diego.bottegal@udl.cat (D.N.B.); javier.alvarez@udl.cat (J.Á.-R.); 2 Instituto Nacional de Tecnología Agropecuaria (INTA), Rivadavia 1439, Ciudad de Buenos Aires C1033AAE, Argentina; 3 Departamento de Producción Animal y Ciencia de los Alimentos, Universidad de Zaragoza, 50013 Zaragoza, Spain; 4 Instituto Agroalimentario de Aragón—IA2, CITA—Universidad de Zaragoza, 50013 Zaragoza, Spain; slobon@cita-aragon.es; 5 Departamento de Ciencia Animal, Centro de Investigación y Tecnología Agroalimentaria de Aragón (CITA), 50059 Zaragoza, Spain; 6 Department of Animal Nutrition and Feed Industry, BonÀrea Agrupa, 25210 Guissona, Spain; marsal.verdu@bonarea.com

**Keywords:** carob pulp, α-tocopherol, condensed tannin, polyphenol, palatability

## Abstract

**Simple Summary:**

To ensure long-term sustainability, the meat industry seeks to apply more sustainable practices like replacing imported traditional feedstuff (i.e., cereals) with low-cost local resources. In this sense, carob pulp (Cp), a Mediterranean by-product rich in polyphenols, represents a potential alternative to feed pigs. Polyphenols exhibit antioxidant properties comparable to vitamin E (Vit E) but may impair animal growth in high doses. This study evaluated the influence of supplementing fattening pig diets with 20% Cp and high doses of Vit E (300 IU/kg) on growth performance, economic results, physiological parameters, nutrient digestibility, and animal behaviour. While no synergistic effect between Cp and Vit E was observed, incorporating 20% Cp in pig diets during the last 40 days of the fattening period is feasible without compromising growth performance, physiology, or behaviour. However, crude protein digestibility and the feeding cost were negatively affected by Cp inclusion. Supplementation with Vit E, although not improving pig performance, increased α-tocopherol deposition in blood, potentially enhancing the antioxidant tissue profile and meat quality.

**Abstract:**

This study aimed to assess the impact on growth, economic results, apparent nutrient digestibility (CTTAD), physiological variables, and animal behaviour when 214 fattening pigs (78 ± 8.5 kg of initial body weight and 130 ± 4.5 days of age) of both sexes (gilts and boars) were fed two levels of carob pulp (Cp, 0 vs. 20%) and two doses of vitamin E (Vit E, 30 vs. 300 IU/kg) for 40 days. No interaction effects between factors studied (Cp, Vit E, and sex) were observed on the variables. Most productive traits were unaffected by Cp or Vit E inclusion. However, the Cp increased the feed conversion ratio during the first 20 days. The Cp group showed a higher CTTAD of ether extract and hemicellulose but lower CTTAD of crude protein. Pigs fed Cp had a lower plasmatic urea content than the control group. The high Vit E doses increased the CTTAD of every nutrient and the plasmatic α-tocopherol content. The pigs fed Cp tended to spend more time eating in the early morning, likely to mitigate tannins’ astringent effects. Dietary inclusion of 20% Cp in finishing high-conformation pigs is possible without affecting overall performance though it reduces nutrient CTTAD and increases feeding cost. Supra-nutritional doses of Vit E do not affect pig performance but increase the α-tocopherol deposition with potential antioxidant effects.

## 1. Introduction

The swine industry faces challenges related to environmental impacts, animal welfare concerns, ethical issues, and rising feeding costs despite its crucial role in providing high-quality animal protein [1,2]. Consequently, the meat industry must adopt more sustainable practices such as optimizing the composition of animal diets [3]. Using agro-industrial by-products may reduce local feed–food competition and boost circular economy principles. Therefore, regional products have been re-explored to replace conventional ingredients, such as cereals, while ensuring pig performance and health. In the Mediterranean area, carob pods and pulp (*Ceratonia siliqua* L.) could be considered among these by-products since they are an ancient resource in dry areas and have significant use in the agri-food industry due to their nutritional value and gum production [4]. In addition, Spain is a major carob producer (47,300 t of carob/year, in 2022 [5]) alongside Portugal, Italy, Morocco, Turkey, and Greece [4], and it has also been the main swine producer in the European Union during recent years [6].

Nutritionally, carob pulp (Cp) is considered a rich source of dietary fibre, sugars, and polyphenolic compounds, mainly condensed tannins (CTs) [7,8]. In pig diets, the use of fibrous and tannin-rich ingredients must be controlled to avoid productive losses; thus, FEDNA guidelines [9] established a maximum incorporation threshold of 60 g/kg of Cp in finishing pigs, which represents 20 g of neutral-detergent fibre/kg of feed. Nevertheless, inclusion levels of up to 250 g/kg of that type of fibre in finishing pig diets does not impair the growth performance [10]. Assessing animal behaviour alongside physiological variables can help us understand the impact of non-conventional ingredients on feed preference and animal health [11]. For instance, the inclusion of fibre in confined pigs may increase chewing activity, production of saliva and gastric juice, delay gastric emptying, and increase the production of volatile fatty acids, which are associated with satiety [12]. Early studies on animal feed polyphenols, such as tannins, highlighted the antinutritional features at high doses, as they can form indigestible complexes with proteins, polysaccharides, and minerals within plant tissue [13]. Indeed, CTs are related with astringency properties, which might affect palatability and consequently feeding behaviour [14]. However, dietary inclusion of moderate tannin levels (<50 g/kg) has been reported to exhibit antioxidant, anti-inflammatory, antipathogenic, and prebiotic properties, which can positively influence gut health, nutrient utilisation, and therefore animal welfare [15,16]. In pigs, those effects have been tested mainly in post-weaning piglets fed with carob by-products [17,18], and only a few studies evaluated their potential interest at low doses (up to 15%) in finishing diets [19,20,21]. However, no effects of Cp polyphenols at those doses were denoted on the physiology or behaviour of fattening pigs. Therefore, it is of interest to investigate whether increasing the Cp dose (e.g., 200 g/kg of feed) may produce additional effects without compromising animal performance. Conversely, higher doses might reveal the antinutritional effects of CTs.

The antioxidant capacity of polyphenolic compounds is extensively researched in the food industry and medicine due to their potential to prevent diseases associated with oxidative stress [22,23]. However, the antioxidant efficacy of these compounds in monogastric diets is still unclear. Furthermore, they may interact with other antioxidant compounds highly used in animal feed, such as α-tocopherol, which is the most active isomer of vitamin E (Vit E). In pigs, the recommended level of Vit E supplementation is 12–25 IU/kg of feed, to avoid muscular pathologies [24]. Nevertheless, supplementing diets with higher doses of Vit E have been associated with improved immunity, oxidative stability of lipids, overall growth performance, and extended shelf life of pork [25,26,27].

The combined dietary inclusion of Cp and high doses of Vit E in pigs may positively impact performance, nutritional physiology, and animal behaviour, leading to improved productivity and animal welfare in pigs reared under intensive conditions. To the best of our knowledge, this is the first study testing the possible interactions between these ingredients in fattening pigs. Therefore, this work aimed to evaluate the effect of the dietary inclusion of 200 g Cp/kg and a high dose of Vit E (300 IU/kg) on growth performance, economic results, nutrient digestibility, blood nutritional and physiological indicators, and the time-budget behavioural pattern of commercial genetic lines of fast-growing pigs.

## 2. Materials and Methods

### 2.1. Animals and Experimental Diets

The protocols employed in this research received approval from the Ethical Committee for Animal Experimentation at the University of Lleida (resolution N° CEEA 02-03/21 procedure N° 02). The animals were reared under standard commercial conditions at the BonÀrea group’s experimental facilities of Nial farm(Guissona, Spain). The rearing practices adhered to the Spanish Animal Protection Regulations RD 53/2013, which align with the guidelines outlined in the European Union Directive 2010/63 for experimental animals’ welfare and ethical treatment.

In this study, a total of 220 crossbred (55% gilts and 45% boars) undocked tail pigs originating from Danbred Duroc sires and Landrace x Large White dams were used. The animals were received with 70 ± 4.5 days of age and 19 ± 4.2 kg of body weight (BW). Once at the facility, they were individually ear-tagged and split-sex allotted to 44 pens (half of them gilts and half entire males) based on initial BW (5 pigs/pen, 1 m^2^/animal) in two similar barns with natural ventilation. All pens had slatted floors with a chain as point-source enrichment, a cup drinker, and individual single-space feeders with dry feeding. The environmental conditions of the facilities were registered by data loggers Elitech RC-51H (Elitech Technology, London, UK). The mean temperature and relative humidity during the experiment were 20.7 ± 2.75 °C and 73.2 ± 9.00%, respectively.

Sixty days before the nutritional experiment began, all animals were fed a standard commercial growing–fattening pelletized diet. During this period, animals were supervised daily and weighed every 21 days. The experimental phase started when the animals reached 130 days of age and 78 ± 8.5 kg of BW, considered the initial age and weight, respectively. Subsequently, each pen was randomly assigned to one of four dietary treatments (11 pens per treatment) for 40 days. The nutritional experiment was designed as a 2 × 2 factorial. The diets presented two inclusion levels of Cp (0 vs. 200 g of Cp/kg of feed) and two doses of Vit E (30 vs. 300 IU/kg of feed); therefore, the diets were defined as 0% Cp-30IU Vit E, 0% Cp-300IU Vit E, 20% Cp-30IU Vit E and 20% Cp-300IU Vit E. The Cp was included maintaining the same ingredients across all diets (Table 1), and the diets were formulated to meet the ideal protein content and to keep a similar net energy (9.7 MJ of net energy/kg of feed) (Table 2). In addition, all diets maintained a constant relationship between the calculated standardized ileal digestible (SID) amino acid and the digestible energy supply in the diets (Table 2). Feeds were provided ad libitum and in pelleted form throughout the fattening period.

On day 169 of age (end of the experiment), and after 18 h of fasting (deprived of feed but not of water), all animals were marked with an individual tattoo number on each side of the body and then loaded carefully (with a hydraulic lifting ramp) onto the truck in small groups (11–12 pigs/pen inside the truck). On the same day, all animals were transported 3 km to the commercial slaughterhouse of BonÀrea Agrupa (La Closa, Guissona, Spain). Once the pigs arrived at the slaughterhouse, they were allocated to three lairage pens (~60 animals/pen and 0.53 m^2^/animal) for 30 min on average. Animals were stunned with 85% CO_2_ for 120 s and immediately exsanguinated.

### 2.2. Feed Analysis

Feed analyses were conducted on samples of the experimental feed taken directly in the factory, once produced, to ensure the consistency between the expected (formulation) and real nutrient compositions. Likewise, subsequent samples were taken throughout the trial (at the middle and the end of the study) to monitor the quality of the diets offered (Table 2). All samples underwent duplicate analyses, following the protocols established by the AOAC [28]. The dry matter (DM) content was determined using the oven drying method (934.01), while the total ash content was measured using a muffle furnace (942.05). Crude protein (CP) levels were assessed using the Kjeldahl method (976.05) with an MT 2300 analyser (Höganäs, Switzerland). For the determination of ether extract (EE), the Ankom Extraction System XTI5 (ANKOM Technology, Fairport, NY, USA) was utilized, with prior hydrolysis. The neutral detergent fibre (aNDFom), with a heat-stable amylase treatment, and acid detergent fibre (ADFom) were analysed subsequently in the same sample using an ANKOM 220 Fiber Analyzer (ANKOM Technology, Fairport, NY, USA) after passing the samples through acetone to remove excess fat. Both aNDFom and ADFom were expressed exclusively as residual ash. The total carbohydrates were estimated as follows: DM − (CP + EE + ash). Total starch was enzymatically analysed using the Total Starch Assay Kit K-TSTA07/11 from Megazyme (Bray, Ireland). Gross energy was determined on feed using a bomb calorimeter (Gallenkamp auto bomb CBA-305-010M; Sanyo Gallenkamp PLC, Sussex, UK).

The α-tocopherol content was analysed by liquid chromatography in 200 mg of the freeze-dried feed samples according to the methodology described by Blanco et al. [29]. After an extraction process, dry residue was obtained, which was dissolved in a mobile phase and injected into an ACQUITY UPLC H-Class liquid chromatograph (Waters, Milford, MA, USA) equipped with a silica-based bonded phase column (Acquity UPLC HSS T3, 1.8 μm × 2.1 mm × 150 mm column, Waters), an absorbance detector (Acquity UPLC Photodiode Array PDA eλ Detector; Waters), and a fluorescence detector (2475 Multi λ Fluorescence Detector, Waters) controlled by the Empower 3 software.

The total polyphenols were extracted both in the freeze-dried feed samples and raw Cp, and later quantified following the Folin–Ciocalteu reaction, according to Rufino-Moya et al. [30]. Samples and standard calibrations were measured with a Heλios β spectrophotometer (Thermo Electron Corporation, Waltham, MA, USA) at 725 nm, and polyphenol contents were expressed as tannic acid equivalents.

The durability of the pelletized particles was assessed using a Pfost durability tester, which calculates the durability index as = 100 × (weight of pellets after 10 min of tumbling at 50 rpm/weight of pellets before tumbling).

The Cp had the following chemical composition: 864 g DM/kg of Cp, 46.5 g of CP/kg of DM, 6.0 g of EE/kg of DM, 34.6 g of ash/kg of DM, 169 g of aNDFom/kg of DM, 153 g of ADFom/kg of DM, and 94.2 g of lignin/kg of DM. In addition, the pulp had 204.3 g Cp total condensed tannins -eq./kg of DM proportionally formed by 15.0% extractable CTs, 82.0% protein-bound CTs, and 3.00% fibre-bound CTs of the total CTs.

### 2.3. Growth Performance

On the days 130, 151, and 169 of age, all animals were individually weighed using an electronic weighing scale. The within-pen coefficient of variation in BW was calculated as follows: 100 × (standard deviation of BW in the pen/average BW in the pen) [16]. The average daily gain (ADG) was calculated for each period and for the overall experiment. At the same time, in each pen, the offered feed and the refusal were also weighed to estimate the average daily feed intake (ADFI). The feed conversion ratio (FCR) was calculated as ADFI/ADG. At the abattoir, the hot carcass weight (unchilled carcass without hands and internal organs) was individually recorded in the slaughter line, and then the dressing percentage was calculated as follows: 100 × hot carcass weight/final BW. In the slaughter line, the AutoFom III ultrasonic system (Frontmatec A/C; Herlev, Denmark) was used to estimate lean meat percentage of each carcass.

### 2.4. Economic Evaluation

A sensitivity analysis was conducted to determine the effect of including Cp and Vit E in the diet of pigs (gilts and boars) on the economic benefits. The analysis was established under different scenarios based on the available information on raw material and pork carcass prices, from week 11 to 52 of 2022 [31,32]. Moreover, the scenarios combined minimum and/or maximum feed costs and income during that period.

The price of each diet (EUR/kg feed) was calculated weekly considering fluctuations in the prices of the ingredients, informed by the MIAVIT Animal Nutrition S.L company (Tarragona, Spain). The chopped Cp prices were taken from economic reports of the Department of Agriculture and Livestock of Catalunya, Spain [33]. The lowest, average, and highest Cp prices were 220, 260, and 270 EUR/t of Cp, respectively.

Likewise, a value of 2152 EUR/t was set for the mixture of the vitamin–mineral premix and added macro-minerals (Na, P, Ca). The total feeding cost was calculated considering the partial cost of each of the feeding phases: nursery (from 6 to 18 kg of BW), growing phase (from 18 to 78.4 kg of BW), and experimental fattening phase (from 78.4 to 125 kg of BW). Feed consumption for the nursery and growing phases was set at 18.9 and 144 kg of feed/animal, respectively.

Income, expressed as EUR/kg of BW, was calculated as follows: [carcass price (EUR/kg) × hot carcass weight]/BW at slaughter. The carcass price was defined by the lean level and was classified as class S (>60% lean) or E (55–60% lean) [32].

The scenario with the highest total feeding cost (1.04 EUR/kg of pig sold) was defined between March and June, while the lowest cost (0.97 EUR/kg of pig sold) occurred between November and December. Minimum or maximum income scenarios were established considering the price per kg of S-class carcasses ranging from 2.09 EUR to 2.19 EUR/kg of carcass, respectively. The scenarios were categorized as minimum income–minimum cost, minimum income–maximum cost, maximum income–maximum cost, and maximum income–minimum cost.

### 2.5. Faecal Sampling and Estimation of Apparent Digestibility of Nutrients

On day 166 of age, faecal samples (approx. 100 g/pig) were collected from three animals per pen through rectal stimulation, to assess the consistency and apparent digestibility of nutrients. After collection, faecal samples were dried for 7 days at 60 °C (until they reached stable weight) and stored at −20 °C. After thawing, faecal samples from each pig were ground in a knife mill (1 mm sieve). To perform proximate chemical analyses (DM, organic matter -OM-, ash, CP, and EE), these samples were pooled with those from other pigs from the same pen, creating a representative sample. In addition, the hemicellulose content was calculated as the difference between aNDFom and ADFom. The acid-insoluble ash (AIA) was considered a natural marker, and its content was determined following the method described by Sales and Janssens [34], which consisted of drying, ashing, and boiling the ash in hydrochloric acid. Then, the ashes were filtered and washed with hot water and re-ashed. The coefficient of apparent digestibility (CTTAD) was estimated based on the ratios of faeces and feed AIA, as follows:

CTTAD = 1 − ((AIAd/AIAf) × (Zf/Zd)),

where Zf and Zd are the nutrient concentrations (g/100 g) in the faeces and the diet, respectively, and AIAd and AIAf are the concentrations of acid-insoluble ashes (g/100 g) in the diet and the faeces, respectively.

### 2.6. Blood Samples, Nutritional and Oxidative State Markers

One animal per pen was randomly selected and bled (a total of 44 animals) by trained personnel on day 166 of age through jugular venipuncture, using 1.2 × 38 mm needles and 10 mL Vacutainer tubes with EDTA as an anticoagulant (BD^®^ Vacutainer^®^ (Plymouth, UK) with EDTA K2 additive). Each tube was carefully turned after the blood sample was taken to allow the blood to interact with the anticoagulant. The haematocrit (expressed in %) was manually determined by duplicate after centrifugation of a microcapillary tube filled with whole blood [35]. Subsequently, to separate the plasma, the tubes were centrifuged (Cencom II angular rotor centrifuge, JP Selecta^TM^, Abrera, Spain) at 3000 rpm for 15 min. A 1.5 mL aliquot of plasma was transferred to Eppendorf tubes and stored at −80 °C until further analysis. Plasma urea, creatinine, glucose, lactate, triglycerides, non-esterified fatty acids (NEFA), and cholesterol were analysed by an automatic analyser Olympus AU4000 series 3112676 (Hamburg, Germany). All protocols and reagents were standardized and provided by the Biochemical Veterinary Laboratory (Autonomous University of Barcelona, Barcelona, Spain). The mean intra- and inter-assay coefficients of variation for these metabolites were 1.22% and 2.35%, respectively.

The plasma α-tocopherol concentration was analysed using the same high-performance liquid chromatography (HPLC) process as the feed α-tocopherol analysis, following the methodology described by Rufino-Moya et al. [36]. Briefly, 1 mL of plasma and 1 mL of ethanol were mixed and shaken in a 15 mL polypropylene tube for 1 min. Then, the lipophilic compounds were extracted twice; thus, 5 mL of hexane:ethyl acetate (9:1, *v*/*v*, containing 5 μg/mL of BHT) solution were added. The mixture was shaken on a rotary mixer for 10 min and then centrifuged for 5 min at 3500 rpm and 10 °C. Each time, the upper phase was transferred into a 12 mL amber glass tube. The sample was vacuum evaporated in a rotary evaporator for 30–40 min at 40 °C and then the residue was resuspended in 1 mL of mobile phase (acetonitrile:methanol:dichloromethane, 75:15:10, *v*/*v*/*v*). This mixture was vortexed and shaken on a rotary mixer for 10 min. Finally, the suspension was taken and collected through a syringe with a filter in a 2 mL amber vial for HPLC analysis.

A ratio between α-tocopherol and lipid contents was calculated as follows: α-tocopherol (mg/L)/sum of cholesterol (g/L) and triglycerides in plasma (g/L) [37].

The oxidative state of the plasma was assessed through the malondialdehyde (MDA) concentration, which was determined using the method described by Yonny et al. [38] and was expressed as the sum of the free MDA and protein-bound MDA, which were quantified separately.

### 2.7. Animal Behaviour and Health Measurements

The animal behaviour was recorded through the instantaneous scan sampling technique four times during the study (every 9 days), following the methodology described by Argemí-Armengol et al. [39]. Each day, two trained observers, one per barn, carried out direct observations on 16 pens/barn (2 pens/diet/sex) Each pen was scanned every 10 min during three daily sessions of 1.5 h in length (7:30–9:00, 10:00–11:30, and 12:30–14:00, designated as early morning, mid-morning, and midday, respectively). Thus, 10 observations were performed per pen per session. To minimize observer influence, they entered the barn and walked around the animals for 30 min, before starting the observation. During each observation (around 30 s), the observer recorded the number of pigs per pen displaying some of the behaviours or postures previously established (Table 3) before moving calmly to the next pen. Therefore, the frequency of each behaviour was calculated as follows: (number of pigs demonstrating that behaviour/total number of pigs in the pen) × 100. Ten minutes were sufficient to thoroughly observe all pens and restart the cycle.

All pigs were assessed for skin lesions and tear staining at the end of the trial. Skin lesions were visually assessed in five separate regions (ears, neck, middle, hindquarters, legs) on the left side of each pig at a distance of 0.5 m following the methodology proposed by Welfare Quality [41]. Furthermore, lesions were scored on a 0 to 2 scale (0 = up to 4 lesions, 1 = 5–10 lesions, and 2 = more than 11 lesions) and their prevalence was determined as a percentage of score 1 and of score 2 by pen [42]. In addition, the tear staining was measured on the left eye following the methodology of Telkänranta et al. [43], but the score scale was modified to facilitate evaluation: 0, no signs of staining; 1, staining detectable and the stained area is lower than 50% of the total eye area; 2, staining easily detectable, may exceed the 100% of total eye area but does not extend below mouth; and 3, the stained area extends below the mouth line. The tear staining index, which considered both the frequency and the severity of the staining (ranged from 0 to 300, where 0 is absence and 300 is for all animals with severe tear staining), was calculated as follows: score 1 (%) + 2 × score 2 (%) + 3 × score 3 (%) [44].

### 2.8. Statistical Analysis

Data were analysed with the statistical software Infostat (version 2020, Centro de Transferencia InfoStat, Universidad Nacional de Córdoba, Córdoba, Argentina [45]). The Shapiro–Wilk test was used to examine the normal distribution of the data. The pen was considered the experimental unit for the growth performance, economic results, feed digestibility, physiological variables, and behavioural observations. These data were analysed through standard least square models that included the Cp inclusion level (0 and 20%), Vit E dose (30 and 300 IU), sex (gilts and boars), and their interactions. The clinical measurements (skin lesions and tear staining) did not show normal distributions and were submitted to Kruskal–Wallis analyses. The significant level was established at *p* ≤ 0.05 and differences among means with 0.5 < *p* < 0.10 were considered as tendencies to difference. In all cases, single interactions between fixed effects were removed from the final model where non-significant effects were found (*p* > 0.05). Nevertheless, if significant interactions were detected, a Tukey test was performed to separate means. The results are expressed as least square means with their associated standard error.

A multivariate canonical discriminant analysis was performed to determine combinations of observed variables (measured at the end of the fattening period and at the abattoir) that optimize the grouping of individuals into the experimental diets. Firstly, a stepwise classification model was built, which reduced the number of explanatory variables and considered significant canonical correlations with a *p* < 0.05. Further, a canonical discrimination procedure was used to ascertain the key point variables that would contribute to a discrimination function of pigs based on the Cp and Vit E dietary content.

## 3. Results

A total of 214 pigs completed the experiment, since six pigs were removed from the trial due to causes unrelated to diet (prolapse, lameness, umbilical hernia, or wasting syndrome) and their data were not included in the study. The culling rate was similar between Cp (2.8 ± 0.03%, *p* = 0.10) and Vit E (3 ± 0.03%, *p* = 0.376) treatments and sex (3.0 ± 0.01%, *p* = 0.273).

No significant interaction was detected between Cp, Vit E, and sex on any variable (*p* > 0.10); therefore, results are shown as main factors in Tables and Figures.

### 3.1. Animal Performance

The effect of the main experimental factors on the growth performance are shown in Table 4.

During the first part of the experimental period (130–151 days of age), neither Cp nor Vit E affected the BW, ADG, or ADFI, but the FCR was 4% higher (*p* = 0.037) in the 20% Cp group than in the 0% Cp group. In that period, boars showed a higher (*p* = 0.002) ADG and tended (*p* = 0.096) to have a lower FCR than gilts. In the second period (151–169 days of age), the Vit E supplementation had a scare effect on the animal performance, since only the high Vit E tended (*p* = 0.058) to increase the ADG compared with the low Vit E group. Regarding sex, during that phase, boars had a greater ADG (*p* < 0.001) and ADFI (*p* = 0.015) compared with gilts, but no differences were found in the FCR.

Finally, during the whole experimental period, the animal performance was not affected either by Cp or Vit E (*p* > 0.10). Likewise, there were no differences (*p* > 0.10) due to the inclusion of Cp or Vit E on the lean content (60.75 ± 0.20%) nor on the proportion of S carcasses (>60% lean) obtained, which represented 72.5% of the total carcasses. However, as was expected, boars grew faster (*p* < 0.001, +17.4%), ate more feed (*p* = 0.020, +6%), and were more efficient at converting feed into gain (*p* < 0.001) than gilts. Therefore, boars showed greater final BW (*p* = 0.001, +9%) but tended to be less homogeneous within pens (*p* = 0.06) than in the case of gilts. No influence of Cp or Vit E was observed (*p* > 0.01) on the within-pen variation in the final BW. At the slaughterhouse, there were no differences between the dietary inclusion of Cp or Vit E (*p* > 0.10) on carcass parameters. However, carcasses from boars were heavier (*p* = 0.04; +6%) and had a lower dressing percentage (*p* < 0.001; −3%) than carcasses from gilts.

### 3.2. Feed Cost, Incomes, and Economic Benefits

The impacts of the dietary inclusion of Cp and/or high doses of Vit E on economic outcomes of fattening pigs are presented in Table 5. No significant effects (*p* > 0.05) on incomes were detected due to the inclusion of Cp or Vit E. The sex did not affect (*p* > 0.05) the income obtained per kg of pig sold, either. Including 20% of Cp in the diets increased the feeding cost (*p* < 0.05) compared with the 0% Cp diet in every scenario. Conversely, the high level of Vit E increased (*p* < 0.05) the annual average feeding cost compared to the low Vit E group. Additionally, feeding costs were higher for gilts (*p* < 0.001) compared to boars. No differences were detected in the economic benefit due to Vit E or sex, although including 20% Cp in diets tended to (*p* < 0.094) decrease the annual average benefit in comparison to the 0% Cp group.

### 3.3. Apparent Digestibility of Nutrients

The DM of the faeces and apparent digestibility were analysed in the sampling pigs at the end of the experiment (Table 6). Neither Cp, Vit E inclusion, nor sex affected the faeces DM (*p* > 0.10). The pigs fed with the 20% Cp diet had a lower CTTAD of the CP (*p* < 0.001, −7%), and greater CTTAD of EE and hemicellulose (*p* < 0.001, +15 and +18%, respectively) than pigs fed with the 0% Cp diet. Meanwhile, supplementation of high doses of Vit E increased the CTTAD of all nutrients analysed (*p* < 0.05). Furthermore, no effects of sex were found on the CTTAD of any nutrient (*p* > 0.10).

### 3.4. Metabolic and Antioxidant Status Profile

Table 7 shows the effects of the main factors (Cp, Vit E, and sex) on the physiological variables analysed at the end of the study. The animals fed with Cp tended to have a greater haematocrit level (*p* = 0.08) and showed a lower (*p* < 0.01) level of urea than pigs without Cp. No other plasmatic variables were affected by the Cp inclusion (*p* > 0.10). Additionally, the boars showed a decreasing haematocrit level (*p* < 0.05) and lower concentration of lactate (*p* < 0.05), urea (*p* < 0.001), and creatinine (*p* < 0.01) in plasma than gilts. The triglycerides, cholesterol, and NEFA contents in plasma were unaffected by any factor studied (*p* > 0.10).

As expected, the pigs fed with 300 IU Vit E had an increased (*p* < 0.001) plasmatic α-tocopherol content compared to control pigs, but it did not modify the plasma concentration of MDA (*p* > 0.10). The ratio of α-tocopherol to the sum of lipids (cholesterol + triglycerides) remained higher (*p* < 0.001) in the 300 IU Vit E group than in the 30 IU Vit E group. The Cp inclusion or sex did not affect this ratio (*p* > 0.10)

### 3.5. Behavioural Patterns and Clinical Evaluation

**Figure 1:**
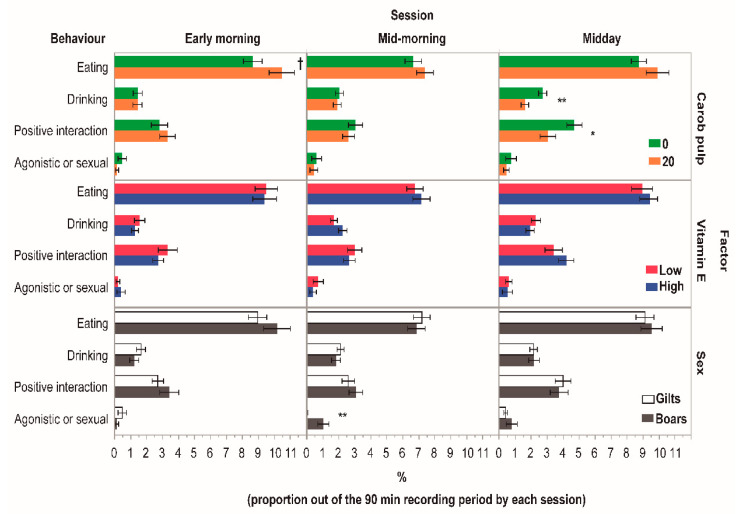
Effects of dietary inclusion of carob pulp (0 vs. 20%) and supra-nutritional doses of vitamin E (30 and 300 IU/kg) on the time budget spent (%) in maintenance (eating and drinking) and social behaviours (positive and agonistic interaction) of fattening pigs (gilts and boars). ^†^, *p* < 0.10; * *p* < 0.05; ** *p* < 0.001.

Figure 1 shows the proportion of maintenance behaviours (eating and drinking) and social behaviours (positive interactions and agonistic or sexual activities) on the activity budget according to experimental factors and daily sessions. In addition, lying (ventral and lateral positions), standing/locomotion, sitting and explorative behaviours were analysed, and data are only shown within the text. Lying was the behaviour in which animals spent the most time, and it was unaffected by diets or sex (*p* > 0.10). Throughout the experiment, lying (considered as the sum of ventral and lateral position) represented 66.3 ± 1.5, 70.3 ± 1.7, and 60.0 ± 1.7% of the time budget in the early morning, mid-morning, and midday, respectively. The explorative behaviour was also unaffected by any factor studied (*p* > 0.10) and represented 12.1 ± 0.68, 11.1 ± 0.95, and 16.8 ± 1.7% of the time budget, in the early morning, mid-morning, and midday sessions, respectively. The sitting behaviour was unaffected by Cp or Vit E during the study (*p* > 0.10), and it represented an average of 3.7 ± 0.51%. However, gilts always devoted more time (*p* < 0.05) in a sitting position than boars, representing 4.80 vs. 3.20 ± 0.50%, 4.00 vs. 2.10 ± 0.11%, and 5.10 vs. 2.70 ± 0.31% of the time budget in the early morning, mid-morning, and midday, respectively.

During the early morning (7:30–9:00 h), the eating behaviour tended (*p* = 0.08) to be greater in the pigs fed with 20% Cp compared to the 0% Cp group. However, no additional impact of Cp was found on the eating behaviour throughout the day. Standing/locomotion activity was unaffected by Cp, Vit E, or sex in this session (*p* > 0.10), and it represented 3.3 ± 1.00%.

At mid-morning (10:00–11:30 h), no influence of Cp and Vit E diets (*p* > 0.10) was found on the activity budget variables. However, boars showed a higher agonistic or sexual activity than gilts (*p* < 0.01). In this session, standing activity was unaffected by diets or sex and represented 3.3 ± 0.50% (*p* > 0.10).

During the third session (midday), the animals fed with Cp increased (*p* = 0.015) their standing/locomotion time (2.70 vs. 4.20 ± 0.43%) but, at the same time, decreased their drinking time (*p* < 0.01) and positive social activities (*p* = 0.023) compared with the pigs from the 0% Cp group. The Vit E and sex did not affect any behavioural variable (*p* > 0.10), except sitting activity which was affected by sex.

No effects were found on the tear staining index due to Cp, Vit E, or sex (101.6 ± 10.2; *p* > 0.10). Neither was the proportion of animals injured with skin lesions per pen affected by any factor (*p* > 0.10; Table 8).

### 3.6. Multivariate Analysis

**Figure 2:**
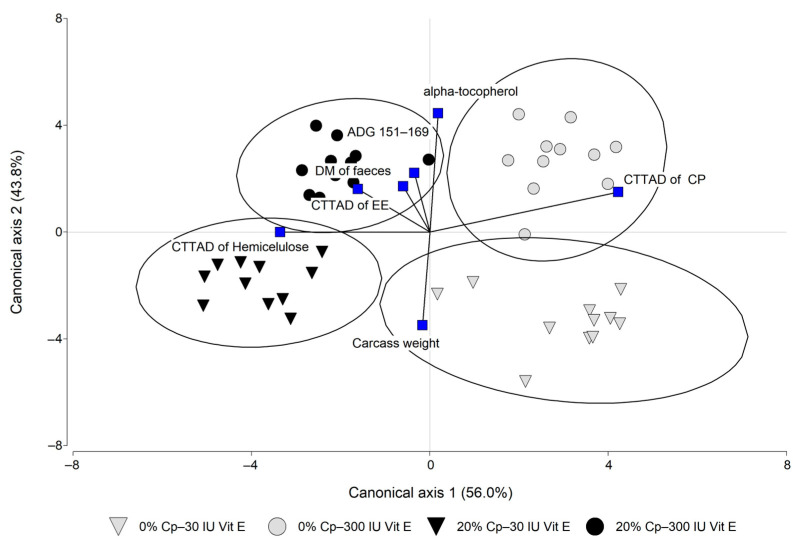
Canonical discriminant analysis of four experimental diets with 0 or 20% carob pulp (Cp) and 30 or 300 IU/kg of vitamin E, indicated as follows: 0% Cp and 30 IU Vit E (grey triangles), 0% Cp and 300 IU Vit E (grey circle), 20% of Cp and 30 IU Vit E (black triangle), and 20% of Cp and 300 IU Vit E (black circle). Variable correlations with the discriminant functions are represented by black straight lines and blue squares. Function 1 (Can 1) accounted for 56.0% of the total variation among feeding strategies and it was determined by the CTTAD of CP (r = 0.72), CTTAD of hemicellulose (r = −0.45) and the CTTAD of EE (r = −0.25). Function 2 (Can 2) accounted for 43.8% of the variance and it was determined by plasma α-tocopherol content (r = 0.78) and carcass weight (r = −0.37). DM, dry matter; ADG 151–169, average daily gain from 151 to 169 days of age.

The stepwise selection procedure reduced variables that may play a role in the differentiation of the dietary experimental groups from 28 to 7. These selected variables were used to classify individuals into four diets on canonical function representations. Canonical discriminant analyses derived canonical variables (Can1 and Can2), which are linear combinations of the quantitative variables that summarize class variation (Figure 2). Function 1 (Can1) discriminated the inclusion of Cp; hence, groups with 20% Cp were assigned to the left quadrants, being related to a greater DM of faeces and higher CTTAD of EE and hemicellulose. In contrast, the 0% Cp groups were consistently located in the right quadrants indicating a higher CTTAD of CP when compared to the other groups. Regarding Function 2 (Can 2), the groups with low doses of Vit E were in the bottom quadrants and were linked to higher carcass weight and lower α-tocopherol plasma content. The high Vit E groups were in the upper quadrant and were related to a higher α-tocopherol plasma content, ADG (151–169 days of age), and CTTAD of CP.

## 4. Discussion

This study aimed to investigate the potential impacts on productivity, digestibility, physiology, and behaviour of fattening pigs when Cp and supra-nutritional doses of vitamin E are included in their diets. Firstly, no interactions between Cp and Vit E were observed in any variable, suggesting that these ingredients affect pigs independently. Studies in broilers [46] and post-weaning piglets [47] showed that dietary combinations of Vit E with different polyphenol sources did not improve animal performance compared to control diets or diets supplemented with only one antioxidant. Even though a synergistic antioxidant effect between secondary plant compounds and tocopherols has been proposed [48], our study could not support this hypothesis in finishing pigs. That effect might probably be verified in animals subjected to stressful situations, such as weaning. However, no acute or chronic stressors were detected in this study; indeed, clinical evaluations (i.e., tear staining and skin lesions) and social behaviour assessments, such as agonistic behaviours, showed no differences between groups. Moreover, no tail-biting events were detected during the trial.

The lack of interaction between dietary treatments and sex indicated that both sexes responded similarly to the evaluated ingredient and additive. Diets were the same for both sexes, so the differences observed were attributable to the effect of sex. Productive advantages (i.e., high feed efficiency) and disadvantages (i.e., low carcass dressing) of fattening boars have been extensively reviewed [49]. Herein, boars showed greater growth performance and carcass weight, which resulted in a lower feeding cost compared with gilts. However, gilts had higher carcass dressing than boars. On the contrary, Vanheukelom et al. [50] found no differences in carcass traits between pigs of both sexes, probably because, in that study, animals were lighter at slaughter than those used in the current experiment. Thus, it is plausible that significant differences in BW emerged with increasing age, as shown in the current study.

The Cp inclusion at low levels (<60 g/kg of feed) in post-weaning piglets’ diets has been considered beneficial to their welfare since it improves intestinal health [17], intestinal ecology [18], and the CTTAD of DM [51]. However, polyphenols, especially tannins, have been related to antinutritional effects during the fattening of high-performance monogastric animals, such as lowering feed intake, growth rate, feed efficiency, and the CTTAD of CP [15,52]. Although some researchers ascertained this concept [53], there are many studies with contradictory results. For instance, Seoni et al. [14] fed boars with up to 150 g/kg of a condensed tannin-rich source (sainfoin) and detected no effect on growth performance compared to the control. In the present study, no harmful impacts of including 200 g/kg of Cp in the diets were found on animal performance during the whole experimental period. Nevertheless, a higher FCR was found in the 20% Cp group during the first 20 days of fattening, which may be due to an adaptation to a slightly more fibrous feed with a higher polyphenol content than the previous. In line with our results, Inserra et al. [20] did not find any effects from including up to 150 g of Cp/kg of feed in fattening pigs’ diets. Similarly, Kotrotsios et al. [19] concluded that a dose of 125 g of Cp/kg had no influence on animal performance compared to the control group. Therefore, the current results suggest that the nutritional requirements were met correctly in both groups (0 and 20% of Cp) by balancing other energy and protein feed sources. Interestingly, the higher ADF and lignin content in the 20% Cp diet did not affect the feed intake compared with the 0% Cp diet.

The economic results of including 20% Cp showed that they depend on the different economic scenarios and the value of the ingredients that Cp might replace. Furthermore, the inclusion of high proportions of some protein and energy sources (e.g., soybean meal and palm oil) within the Cp diet to balance the diets increased the cost of feeding. It is worth noting that the economic study considered the evolution of raw material prices between the 11th and 52nd week of 2022, at which time prices increased significantly compared to previous weeks due to the conflict between Ukraine and Russia. The economic benefit of the group with 20% Cp tended to be lower than the group with 0% Cp, which may be due to the seasonality of carob production and the increase in prices of carob products and by-products after the COVID-19 pandemic. In this sense, the strong demand in the food industry for carob drove the rise in prices [4]. In fact, chopped carob pulp reached the historical maximum of 250 EUR/t (annual average) in 2022, compared to 175 EUR/t in previous years (annual average 2014–2018) [33].

It was suggested that carob’s flavonoids and tannins produce an antidiarrheal effect in piglets because of an increase in the reabsorption of water and electrolytes in the colon [54]. The discriminant analysis linked Cp animals with greater faecal consistency, though no direct validation of these effects was established by the standard least square models.

The Cp inclusion did modify the digestibility of nutrients compared to the control group. The greater inclusion of palm oil in the Cp diets may have improved the CTTAD of EE. The lower CTTAD of CP observed in the Cp groups could be due to the complexes that polyphenols form with proteins, which limit its digestibility. Similar outcomes in the CTTAD of EE and CP were previously observed by Kotrotsios et al. [55] and Beccacia et al. [21] when adding 125 and 150 g/kg of carob pod meal to finishing pig diets. The greatest proportion of CTs in raw Cp were bound to proteins; hence, in the Cp diets, this complex reached around 50% of CTs. Interestingly, the pigs from the Cp group, on average, consumed about 58.9 g of total CTs/day (expressed as g of Cp condensed tannin equivalent) without showing deleterious effects on performance.

Feed palatability as well as digestibility may be impaired by dietary polyphenol content [15]. Nevertheless, the similar feed intake observed between groups may lead to hypothesising that Cp did not impair the palatability of feed. It was suggested that pigs have developed a physiological strategy to counteract the astringency of tannins, which induces hypertrophy of the parotid gland and increases the salivary secretion of proline-rich proteins that bind tannins [56]. Furthermore, pigs fed Cp showed a tendency to spend more time feeding in the early-morning period. During the midday, pigs-fed Cp spent less time drinking and showing less positive social interaction because they spent more time on other activities, such as feeding, although no statistical differences were found. The results suggest that the CTs present in Cp modify the consumption pattern of the pigs with Cp, which must remain longer in the trough or consuming feed, although this is not reflected in differences in the amount of feed ingested per day or in productive performance. These results could benefit animals with restricted feeding, such as gestating sows that quickly consume their daily ration and show signs of anxiety. Previous studies including sainfoin in the finishing diets found that animals visited the feeder fewer times but spent more time and consumed more feed in each visit [14]. These authors suggested that the alteration in the feeding behaviour depended mostly on the CTs content in the sainfoin diets. Similarly with the current study, dietary crude fibre and EE content increased linearly when the CTs source was included. While the increasing fibre was minimal in the present 20% Cp diet compared with the 0% Cp diet, the higher EE content would have positively affected the palatability and impacted feed preferences [57].

Unlike other tannin-rich sources, carob pulp is characterized by its high sugar content, which could activate the sweet receptors in the tongue and improve feed preference [57]. However, the lack of differences in feed intake suggests that the effects of CTs (i.e., astringency or post-prandial regulation of appetite [58]) may have counterbalanced the positive initial sugar effects on palatability.

Regarding Vit E, no differences were detected between doses on growth performance. Upadhaya et al. [59] did not find any effect of the supra-nutritional supplementation of Vit E (300 IU) on any productive parameters in pigs of 6–12 weeks old (i.e., 78–115 kg of BW). Similarly, Niculita et al. [60] did not detect any differences in the final BW of pigs fattened with three different doses of Vit E (11, 100, or 300 IU), although the ADG increased proportionally to the Vit E dosage. In heavy pigs (>145 kg of final BW), supplementing 200–300 IU of Vit E increased ADG slightly, improved FCR in the late stage, and increased hot carcass weight in comparison with the control animals [26,61]. Herein, the slaughter weight (124 kg of final BW) was lower than the mentioned works. However, a tendency to increase ADG during the last 20 days was also recorded in pigs supplemented with 300 IU of Vit E. In line with Upadhaya et al. [59], a positive effect of the high dose of Vit E on the digestibility of all the analysed nutrients was established, which might have increased the above-mentioned performance. Likely, α-tocopherol protects the integrity of the intestinal mucosa; therefore, its consumption improves the absorptive capacity [62]. The greater CTTAD of EE in the high Vit E diets may be explained by considering the fat-soluble nature of the vitamin, since it shares digestive and absorptive mechanisms with other lipids [63]. In accordance with Wang et al. [26], the current results indicate that the level of Vit E recommended by several swine nutritional guidelines [24,64] might be revised to maximize both digestive function and productive performance in heavy pigs.

Most of the physiological variables evaluated herein did not show changes associated with the nutritional treatments. Only the plasma urea concentration decreased in the Cp pigs, which may be explained by the lower CTTAD of CP observed in this group. In all cases, productive results suggest that the contributions of digestible essential amino acids met the physiological needs. Even, it would be possible to reduce the CP supplied in diets without affecting the performance of fast-growing fattening pigs. Similarly, gilts showed a higher content of circulating urea than boars. Likely, reducing CP in the gilts diet and formulating diets according to sex is an alternative to reduce nitrogen losses and feeding costs. Pérez-Ciria et al. [65] demonstrated that gilts can be successfully fed diets with 125 g of CP/kg instead of 140 g of CP/kg (providing at least 5.4 g SID Lys/kg), with no adverse effects on their productive performance.

The higher and the longer the supplementation of Vit E, the greater the α-tocopherol muscular deposition [66]. Thus, pigs supplemented with 300 IU of Vit E increased the level of plasma α-tocopherol by 1.9 times in comparison with the pigs that received 30 IU of Vit E. Also, Niculita et al. [60] saw a 2.8-fold increase in the level of α-tocopherol in the serum of animals supplemented with 300 IU of Vit E compared to the control group (11 IU Vit E).

It is known that Vit E supplementation may reduce oxidative stress and subsequently improve pork quality [67]. Dietary polyphenols may also improve the antioxidative status and ameliorate oxidative stress in pigs [52]. However, the plasma MDA content, which is an indicator of lipid oxidation, remained stable in all groups. Considering the lack of pro-oxidative conditions during the fattening period in our study it was not possible to attribute an antioxidant effect to the inclusion of Cp or Vit E.

Some clinical studies, especially in humans, considered that the α-tocopherol–lipid (triglycerides + cholesterol) ratio is an accurate indicator to determine if the current circulating level is optimal [37]. Vitamin E is fat-soluble; therefore, it circulates linked to low-density lipoproteins and its absorption may be enhanced by other dietary lipid components [68]. In the current study, the pigs fed with a high dose of Vit E showed the highest α-tocopherol–lipid ratio but, at the same time, there were no differences between groups in the plasmatic lipid levels (such as cholesterol, triglycerides, and NEFA). Interestingly, Corino et al. [61] reported that heavy pigs with an α-tocopherol–lipid ratio higher than 2.3 showed a dressing percentage 2.1% greater than animals with a low ratio.

Regarding social interaction, boars showed a higher prevalence of fighting behaviour and lower inactive behaviours, such as sitting, in comparison to gilts. These results were expected, as it is known that the presence of testicular hormones in boars increases aggression and sexual behaviour [50]. Nowadays, the use of entire males in farms is an increasing practice; thus, Bee et al. [69] hypothesized that the inclusion of tannin sources before the onset of puberty might suppress or decrease the androgenic activity of the testes. While these hormones are responsible for aggressive behaviour in entire males, introducing Cp as a CT-dietary source did not appear to affect boars’ social behaviour.

## 5. Conclusions

The hypothesis of a synergistic effect between Cp and Vit E inclusion on growth performance and metabolic status of fattening pigs was rejected. Replacing traditional ingredients, mainly barley, with up to 200 g Cp/kg in the diet of high-performance pigs is feasible without affecting productive performance, metabolic status, behaviour, and health measurements. However, including a high level of carob pulp requires careful evaluation due to carob market fluctuations and increased prices. Although the CTs in Cp may exert a negative effect on the CTTAD of CP, the high content of sugar may counterbalance the effects on feed intake. Supplementing 300 IU of Vit E for 40 days does not affect the productive parameters, behaviour, and health status of fattening pigs, although it increases their plasma circulation of α-tocopherol. Further research is needed to assess whether a higher α-tocopherol deposition may improve meat quality and shelf life of meat from pigs fed Cp and/or high-dose Vit E.

## Figures and Tables

**Table 1:** Ingredients of the experimental diets (g/kg, as-fed basis).

Item	Carob Pulp 0%−30 IU Vit E	Carob Pulp 0%−300 IU Vit E	Carob Pulp 20%−30 IU Vit E	Carob Pulp 20%−300 IU Vit E
Corn	163		179	
Barley 9.6% CP	549		290	
Wheat 11.2% CP	70		70	
Soymeal 47% CP	91		158	
Sunflower meal 28% CP	80		39	
Carob pulp	−		200	
Palm oil	15.1		41	
L-Lysine sulphate, 75% ^1^	4.8		3.2	
Hydroxy methionine ^2^	0.2		0.5	
L-Threonine ^3^	0.9		0.7	
Fine calcium carbonate	11.2		3.6	
Dicalcium phosphate	6.1		7.3	
Salt	5.5		4.5	
Premix ^4^	2.7		2.7	
Vitamin E 50% ^5^	−	0.5	−	0.5

^1^ (EU 3c325) L-lysine sulphate produced by fermentation with *Corynebacterium glutamicum*, 52% pure. ^2^ (EU 3c307) Methionine hydroxy analogue, 88% purity. ^3^ (EU 3c410) L-threonine, 98% purity. ^4^ Supplied the following (per kg of feed): 5998 IU of vitamin A, 1498 IU of vitamin D3, 15 IU of vitamin E (all-rac-α -tocopheryl acetate), 5 ppm of B4 (choline chloride), 100 ppm of Fe (iron sulphate monohydrate), 0.3 ppm of I (potassium iodide), 18 ppm of Cu (pentahydrate cupric sulphate), 40 ppm of Mn (manganese oxide), 94 ppm of Zn, 0.34 ppm Se (sodium selenite), 50 ppm BHT (E321), 6 ppm propyl gallate (E310), 500 FYT 6-phytase EC 3.1.3.26, 1220 units endo-1,4-β-xylanase EC 3.2.1.8., 152 units endo-1,3(4)-β-glucanase EC3.2.1.6. ^5^ Cuxavit E 50%, vitamin E (all-rac-α-tocopheryl acetate 285 IU).

**Table 2:** Chemical composition (g/kg, as-fed basis, unless otherwise indicated) and physical pellet quality of the experimental feeds.

Item	Carob Pulp 0%−30 IU Vit E	Carob Pulp 0%−300 IU Vit E	Carob Pulp 20%−30 IU Vit E	Carob Pulp 20%−300 IU Vit E
*Analysed nutrients*				
Dry matter	903		907	
Gross energy (MJ/kg)	17.0		17.6	
Crude Protein	144		153	
Ether extract	37.3		60.9	
aNDFom	130		129	
ADFom	75.0		92.0	
Ash	41.6		44.5	
Lignin	26.1		37.5	
Starch	448		329	
Total sugars	23.2		81.6	
Lysine	8.5		9.1	
Methionine	2.4		2.6	
Threonine	6.2		6.8	
Tryptophan	1.4		1.9	
Isoleucine	5.7		6.2	
Valine	6.6		7.3	
Total polyphenols (g tannic acid equivalents/kg)	5.2		7.8	
Total condensed tannins (g carob pulp internal total CT-eq./kg)	3.6		19.0	
α-tocopherol (g/kg)	31.4	299	30.6	296
*Calculated nutrients*				
Net energy (MJ/kg) ^1^	9.8		9.7	
Total carbohydrates ^2^	653		624	
SID ^3^ Lys	7.5		7.8	
SID Met	2.1		2.2	
SID Thr	5.3		5.6	
SID Trp	1.1		1.5	
SID Ile	4.8		5.0	
SID Val	5.6		5.7	
Durability index (%)	98.1		95.3	

^1^ Net energy estimated according to FEDNA [9] from the ingredient composition of the feeds. ^2^ Total carbohydrates were calculated as DM − (CP + EE + ash). ^3^ Standardized ileal digestibility (SID) of amino acids.

**Table 3:** List of assessed behaviours, adapted from previous studies [12,39,40].

Behaviour	Description
Eating	Animal with the head or snout over the feeder, interacting with the feeder or chewing feed.
Drinking	Animal with the head or snout over the drinking trough.
Positive social	Suckling or scratching some part of the body of another partner or one’s own without producing injuries or a negative response.
Agonistic or sexual	Head or snout in aggressive contact with another pig, negative social behaviour. Also mounting between pigs was recorded.
Lying down (Ventral)	Pig with body recumbent on the sternum or its belly.
Lying down (Lateral)	Animal with body recumbent on the side, which represents a state of greater inactivity due to the total absence of activity and decreased alertness, may reflect a condition of satiety.
Standing or locomotion	Pig is upright on all four legs and can move or not around the pen.
Sitting	Pig is upright on two front legs, and with the caudal end of the body contacting the floor (sitting in a dog position).
Exploring	Pig is nuzzling, sniffing, or chewing any part of the pen.

**Table 4:** Effects of dietary inclusion of carob pulp (Cp, 0 vs. 20%) and vitamin E (Vit E, 30 vs. 300 IU) in finishing diets on body weight (BW), average daily gain (ADG), average daily feed intake (ADFI), feed conversion ratio (FCR), and carcass parameters of pigs (gilts and boars).

Variable	Cp		Vit E		Sex			*p*-Value ^2^		
	0%	20%	Low	High	Gilts	Boars	SEM ^1^	Cp	Vit E	Sex
n of pens	22	22	22	22	22	22				
*BW (kg)*										
Initial ^3^	78.2	78.6	78.9	78.0	76.8	80.0	1.94	0.886	0.762	0.250
Intermediate ^4^	103.3	103.0	104.0	102.3	100.5	105.8	2.10	0.903	0.571	0.090
At slaughter ^5^	125.2	124.2	125.1	124.3	119.4	130.0	2.16	0.759	0.783	0.001
Within-pen coefficient of variation in slaughter BW (%)	9.79	8.25	8.17	9.87	7.91	10.1	0.812	0.185	0.149	0.060
*From 130 to 151 days of age*										
ADG (kg/d)	1.20	1.16	1.20	1.16	1.13	1.23	0.021	0.218	0.163	0.002
ADFI (kg/d)	2.86	2.88	2.89	2.85	2.80	2.94	0.060	0.840	0.640	0.104
FCR	2.40	2.49	2.41	2.47	2.48	2.40	0.031	0.037	0.212	0.096
*From 151 to 169 days of age*										
ADG (kg/d)	1.13	1.18	1.09	1.22	1.04	1.27	0.045	0.363	0.058	0.001
ADFI (kg/d)	3.30	3.36	3.27	3.39	3.23	3.43	0.056	0.471	0.151	0.015
FCR	3.35	2.89	3.42	2.82	3.14	3.09	0.330	0.324	0.212	0.918
*From 130 to 169 days of age–overall*										
ADG (kg/d)	1.20	1.17	1.19	1.18	1.09	1.28	0.021	0.390	0.789	<0.001
ADFI (kg/d)	3.06	3.10	3.07	3.10	3.00	3.17	0.049	0.594	0.649	0.020
FCR	2.58	2.66	2.60	2.64	2.76	2.48	0.033	0.124	0.460	<0.001
*Carcass measures at the slaughterhouse*										
Hot carcass weight (kg)	92.1	91.3	92.3	91.2	89.1	94.4	1.78	0.762	0.661	0.041
Carcass dressing (%)	73.7	73.5	73.7	73.4	74.5	72.6	0.01	0.628	0.554	<0.001

^1^ Standard error of the mean. ^2^ Interactions between factors were tested but non-significant effects were found (*p* > 0.10); thus, results are shown as main factors. ^3^ BW at 130 days of age. ^4^ BW at 151 days of age. ^5^ BW at 169 days of age.

**Table 5:** Supplementation of carob pulp (Cp) and vitamin E (Vit E, 30 vs. 300 IU/kg) in the finishing diet of pigs (gilts and boars) on income, feeding cost, and economic benefit.

		Cp		Vit E		Sex			*p*-Value ^2^		
Variable	Scenario ^4^	0%	20%	Low	High	Gilts	Boars	SEM ^1^	Cp	Vit E	Sex
Incomes (EUR/kg) ^1^	Low	1.51	1.51	1.51	1.51	1.52	1.50	0.016	0.831	0.871	0.193
	High	1.59	1.58	1.59	1.58	1.60	1.57	0.016	0.844	0.856	0.240
	Annual average	1.55	1.54	1.55	1.54	1.56	1.53	0.016	0.832	0.869	0.199
Feeding cost (EUR/kg) ^2^	Low	0.95	0.97	0.96	0.97	0.98	0.95	0.006	0.006	0.184	<0.001
	High	1.05	1.07	1.06	1.07	1.08	1.04	0.006	0.029	0.178	<0.001
	Annual average	0.95	0.99	0.96	0.99	0.99	0.95	0.007	0.001	0.013	<0.001
Benefit (EUR/kg) ^3^	Low I:Low C	0.56	0.53	0.55	0.54	0.54	0.55	0.016	0.218	0.521	0.859
	Low I:High C	0.46	0.43	0.46	0.44	0.44	0.45	0.016	0.277	0.489	0.752
	High I:Low C	0.64	0.61	0.63	0.61	0.62	0.62	0.016	0.238	0.52	0.809
	High I:High C	0.53	0.51	0.53	0.51	0.52	0.53	0.017	0.298	0.489	0.708
	Annual average	0.59	0.55	0.59	0.56	0.57	0.58	0.017	0.094	0.222	0.671

^1^ Income (EUR/kg of pig sold): [carcass price (EUR/kg carcass) × average carcass weight]/BW at slaughter. ^2^ Feeding cost expressed as EUR/kg of pig sold; it was calculated as the sum of the feeding cost of the nursery phase (6–18 kg BW), growing phase (18–78 kg BW), and the fattening experimental phase (78–125 kg BW) during 2022 [32]. ^3^ Economic benefit is expressed as income (I, low or high)—feeding costs (C, low and high). ^4^ The low-income scenario (March–June 2022) and the high-income scenario (November–December 2022) were established based on the prices per kg of the S-class carcass, which were 2.09 EUR and 2.19 EUR/kg, respectively.

**Table 6:** Effect of two dietary inclusion levels of Cp (0 vs. 20%), two doses of Vit E (30 vs. 300 IU/kg of feed), and sex (gilts or boars) on dry matter content (DM) of faeces, coefficients of apparent digestibility (CTTAD) of the organic matter (OM), crude protein (CP), ether extract (EE), and hemicellulose of fattening pigs at 166 days of age (end of the fattening period).

	Cp		Vit E		Sex			*p*-Value ^2^		
Variable	0%	20%	Low	High	Gilts	Boars	SEM ^1^	Cp	Vit E	Sex
DM of faeces (g/kg)	252	294	263	283	285	261	21.5	0.178	0.511	0.430
CTTAD of OM	0.82	0.81	0.80	0.82	0.81	0.81	0.003	0.100	<0.001	0.490
CTTAD of CP	0.77	0.72	0.73	0.76	0.74	0.75	0.004	<0.001	<0.001	0.240
CTTAD of EE	0.61	0.70	0.64	0.67	0.65	0.66	0.009	<0.001	0.030	0.580
CTTAD of hemicellulose	0.34	0.40	0.35	0.39	0.37	0.37	0.009	<0.001	<0.001	0.820

^1^ Standard error of the means. ^2^ Interactions between factors were tested but non-significant effects were found (*p* > 0.10); thus, results are shown as main factors.

**Table 7:** Effects of the dietary inclusion of Cp (0 vs. 20%) and supra-nutritional doses of Vit E (30 vs. 300 IU/kg) in fattening pigs (gilts and boars) at the end (166 days of age) of the experimental period on metabolic and antioxidant status profile.

	Cp		Vit E		Sex			*p*-Value ^2^		
Variable	0%	20%	Low	High	Gilts	Boars	SEM ^1^	Cp	Vit E	Sex
Haematocrit (%)	40.9	42.7	41.5	42.1	42.9	40.8	0.72	0.080	0.584	0.044
Lactate (mg/dL)	2.62	2.46	2.44	2.64	2.89	2.19	0.244	0.642	0.560	0.047
Glucose (mg/dL)	97.0	97.2	99.3	94.9	95.1	99.0	1.94	0.938	0.113	0.162
Urea (mg/dL)	18.6	15.3	16.5	17.4	20.1	13.8	0.91	0.015	0.449	<0.001
Creatinine (mg/dL)	1.50	1.47	1.49	1.48	1.55	1.42	0.031	0.510	0.818	<0.01
Triglycerides (mg/dL)	30.2	28.6	29.0	29.9	29.3	29.5	2.14	0.596	0.763	0.941
Cholesterol (mg/dL)	109	113	111	111	113	109	3.2	0.336	0.982	0.395
NEFA (mmol/L) ^3^	0.08	0.06	0.07	0.07	0.06	0.08	0.015	0.243	0.863	0.236
MDA (µmol/L) ^4^	7.97	8.10	7.95	8.11	7.92	8.15	0.220	0.671	0.612	0.456
α-tocopherol (μg/mL)	3.07	3.09	2.08	4.08	3.14	3.02	0.175	0.915	<0.001	0.613
α-tocopherol (mg):triglycerides + cholesterol (g)	40.9	42.7	41.5	42.1	42.9	40.8	0.72	0.837	<0.001	0.781

^1^ Standard error of the mean. ^2^ Interactions between factors were tested but non-significant effects were found (*p* > 0.10); thus, results are shown as main factors. ^3^ Non-esterified fatty acids. ^4^ Malondialdehyde.

**Table 8:** Effect of supplementation with Cp (0 vs. 20%), supra-nutritional doses of vitamin E (30 vs. 300 IU/kg), and sex (gilts and boars) on the incidence of skin lesions. The results represent the proportion of animals injured by the factor assessed.

Variables (%) ^1^	Cp		Vit E		Sex			*p*-Value ^2^		
	0%	20%	Low	High	Gilts	Boars	SEM	Cp	Vit E	Sex
Pigs with skin lesions	4.10	5.50	4.50	5.00	6.40	3.20	4.50	0.782	0.754	0.268
Pigs with moderate skin lesions	3.18	4.55	4.00	4.00	4.55	3.18	3.88	0.922	0.968	0.443
Pigs with serious skin lesions	0.91	0.91	1.00	1.00	1.82	0.10	0.88	0.999	0.999	0.152

^1^ Data represent the proportion of pigs per pen with lesions; data obtained via the methodology of Welfare Quality [41]. ^2^ Interactions between factors were tested, but non-significant effects were found (*p* > 0.10).

## Data Availability

Data presented in this study are available upon request from the corresponding author.
